# Study of Graphene-Based Strain Sensing Output Signals Under External Electromagnetic Interference Conditions

**DOI:** 10.3390/nano16090509

**Published:** 2026-04-23

**Authors:** Furong Kang, Shuqi Han, Kaixi Bi, Jian He, Xiujian Chou

**Affiliations:** 1 School of Instrument Science and Technology, North University of China, Taiyuan 030051, China; sz202406141@st.nuc.edu.cn (F.K.); b20230614@st.nuc.edu.cn (S.H.); 2 School of Semiconductors and Physics, North University of China, Taiyuan 030051, China; bikaixi@nuc.edu.cn; 3 State Key Laboratory of Extreme Environment Optoelectronic Dynamic Measurement Technology and Instrument, North University of China, Taiyuan 030051, China; chouxiujian@nuc.edu.cn

**Keywords:** terms-electromagnetic interference environments, graphene-based pressure sensor, current density of graphene, baseline drift

## Abstract

Graphene possesses exceptional mechanical strength, high electrical conductivity, and a stable lattice structure, making it an ideal material for sensors in advanced manufacturing. However, these sensors face stability challenges due to complex electromagnetic interference (EMI) environments generated by electrical equipment. Therefore, investigating the influence of EMI on sensor performance is of significant importance. In this study, simulations were performed to analyze electrical parameter perturbations of intrinsic graphene films under EMI conditions. The Magnetic Fields, Solid Mechanics, and Electrostatics modules in COMSOL Multiphysics were employed to construct a coupled model of a three-phase power transformer and a graphene-based pressure sensor. The results indicate that EMI can induce baseline drift on the order of ~5% full scale (FS) in the graphene current density, accompanied by degradation in signal-to-noise ratio (SNR) exceeding ~15 dB under typical simulation conditions. Graphene in direct contact with metal electrodes shows enhanced sensitivity to EMI, with more pronounced noise amplification due to interfacial coupling effects. In contrast, cavity-suspended graphene configurations exhibit relatively improved robustness, suggesting that suspended membrane architectures can mitigate EMI by reducing parasitic coupling and enhancing mechanical isolation. Compared with previous studies, this work highlights the role of multiphysics coupling and membrane suspension in influencing EMI-induced perturbations, providing theoretical guidance for the design of graphene-based sensors in power system and industrial Internet of Things (IoT) applications.

## 1. Introduction

Graphene possesses extraordinary mechanical and electrical properties [1,2], along with a stable lattice structure, making it an ideal material for pressure sensors. Graphene exhibits a Young’s modulus of approximately 1 TPa [3,4], a maximum tensile strain of up to 20% [5], and an exceptionally high carrier mobility rate of 15,000 cm^2^/(V·s) at room temperature [6]. These properties drive the widespread use of graphene structures fabricated via cavity-based silicon dioxide substrate transfer and suspension in pressure sensor development [7,8]. These sensors are widely applied in various fields [9], including environmental monitoring [10], healthcare and biosensors [11], and the automotive industry [12,13].

J. Scott Bunch et al. from Cornell University have demonstrated the first model of a suspended graphene pressure sensor, fabricated by mechanically exfoliating single- and multilayer graphene sheets from graphite onto silicon oxide trenches [14]. M. C. Lemme demonstrated the piezoresistive effect in graphene using a nanoelectromechanical membrane configuration, enabling direct electrical readout of pressure-to-strain transduction [15]. Compared to conventional pressure sensors, these graphene-based sensors exhibit orders of magnitude higher sensitivity per unit area. Tian-Ling Ren developed a graphene-paper pressure sensor that achieves an optimal balance between sensitivity and operational range, making it particularly suitable for wearable applications [16].

The application of graphene sensors holds significant potential to elevate the intelligence of advanced manufacturing industry [17,18]. Nevertheless, the intricate electromagnetic interference (EMI) conditions endemic to industrial settings adversely affect the operational stability and measurement fidelity of sensors [19]. EMI sources prevalent in industrial environment—such as transformers, motors, and power electronics—expose sensors to complex electromagnetic conditions [20]. Sensitive devices, including smart sensors [21], electronic controllers [21], and actuators [22] in advanced manufacturing industry, are particularly vulnerable to such interference. This susceptibility can result in signal drift, reduced SNR, and unstable baselines in sensors [23]. Consequently, studying the signal output of sensors in EMI environments has attracted numerous experimental and theoretical studies. Bondarenko et al. developed a measurement data-driven inverter modeling method capable of accurately pinpointing frequency bands where radiated emissions exceed regulatory limits. This approach provides a theoretical foundation for establishing interference source identification models, enabling targeted mitigation of electromagnetic noise in inverter systems [24]. Jia et al. conducted a simulation-based analysis of how high-voltage cable types and lengths influence common-mode noise in motor drive systems by developing a system-level conducted EMI model. This model provides a comprehensive framework for characterizing electromagnetic interference environments in such systems [25]. Zhai et al. proposed a mitigation scheme to suppress switching devices’ conducted emissions through the integration of DC bus common-mode chokes and EMI filters. Leveraging electromagnetic interference simulation data, their approach enhances the electromagnetic compatibility (EMC) of power switching systems [26]. Technologies such as shielding, filtering, and grounding for EMI control are now highly mature. However, research on the response characteristics of emerging sensors, such as graphene-based sensors, to electromagnetic interference (EMI) is still in its early stages, with many critical aspects yet to be investigated.

Recent advancements in the field of sensors under electromagnetic interference (EMI) environments have highlighted the critical importance of ensuring reliable signal output amidst complex electromagnetic conditions [27]. Research has increasingly focused on understanding the mechanisms by which EMI affects sensor performance, particularly in high-stakes applications such as industrial automation and smart manufacturing [28]. Despite significant progress, several bottlenecks remain, including the need for enhanced shielding techniques and improved sensor designs that can withstand EMI without compromising measurement fidelity [29]. The susceptibility of sensors to EMI-induced noise and signal distortion continues to be a prominent challenge, necessitating further exploration into innovative materials and architectures that can mitigate these effects. This area of research has garnered considerable attention, as evidenced by studies that have investigated the integration of advanced materials, such as graphene, to enhance electromagnetic compatibility (EMC) and minimize interference [30,31]. By addressing these challenges, our research aims to contribute to the development of graphene-based pressure sensors that maintain stable and accurate outputs in EMI environments, thereby positioning itself at the forefront of current industry trends.

This study investigates the response characteristics of a suspended graphene-based pressure sensor in electromagnetic interference (EMI) environments generated by three-phase power transformers. We analyze the output signals of the sensor under EMI, elucidate the interference mechanisms affecting the intrinsic graphene’s signal integrity, and propose a methodology to enhance the electromagnetic compatibility (EMC) of graphene-based pressure sensors. This study employs electromagnetic simulation tools (COMSOL Multiphysics) to replicate real-world industrial EMI conditions using a three-phase power transformer as a representative interference source. By establishing a quantitative correlation between EMI intensity and sensor signal anomalies, we pioneer a framework for simulating performance parameters of graphene-sensitive membranes in micro-pressure sensors under EMI exposure. The graphene-based pressure sensor exhibited stable baseline behavior, with resistance increasing linearly from 1 kΩ to 1.2 kΩ as pressure rose from 0 to 1 bar under EMI-free conditions; this range shifted to 1.1–1.3 kΩ under EMI conditions. Simulations reveal that a baseline drift (>5% FS) is produced in graphene current density under EMI, which reduces the signal-to-noise ratio (SNR) by more than 15 dB. Also, graphene directly interfaced with metal electrodes demonstrates significant EMI-induced noise amplification. In contrast, cavity-suspended graphene architectures exhibit ~40% higher electromagnetic interference (EMI) resilience and improved electromagnetic compatibility (EMC). These findings demonstrate that incorporating a suspended architecture into the sensor membrane significantly enhances electromagnetic compatibility (EMC) by minimizing parasitic coupling and improving mechanical isolation, thereby offering a robust strategy to mitigate electromagnetic interference (EMI) in high-noise applications.

## 2. Modeling and Simulation of EMI Sources and Pressure Sensor

**Figure 1:**
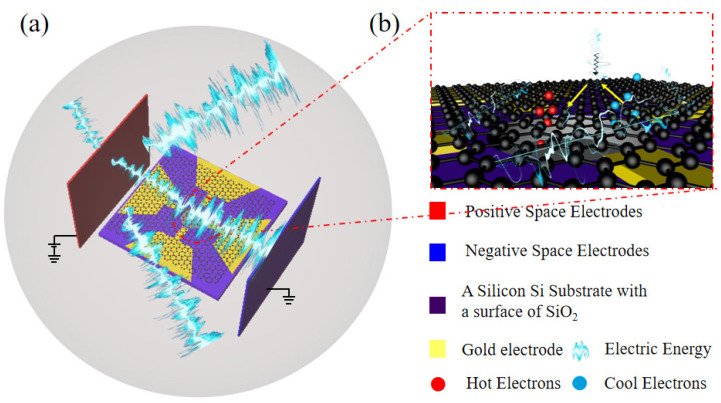
The concept of EMI and pressure sensor. (**a**) Schematic illustration of a micro-pressure sensor in an EMI environment. (**b**) The process by which electric field energy influences the carrier transport in graphene film.

The methodology for modeling electromagnetic interference (EMI) sources, sensors, and their mutual interactions is detailed below. The concept of a sensor operating in an EMI (electromagnetic interference) environment is illustrated in Figure 1. In Figure 1a, a space electrode is utilized to simulate the EMI environment, and the sensors within this environment are the pressure sensors. Figure 1b depicts the process by which electric field energy influences the carrier transport in graphene in the electromagnetic interference. The modeling of the system’s EMI source is divided into three parts: (1) we study a nanoelectromechanical pressure sensor based on suspended graphene; (2) the operational principle of a three-phase transformer is modeled using the electromagnetic module of COMSOL Multiphysics 6.1, and the simulation results provide the magnitude and direction of the external electromagnetic field at various spatial locations relative to the interference source; (3) the EMI environment of the sensor’s sensitive membrane is constructed by integrating the transformer simulation results with the graphene sensor model. The simulated electric and magnetic field distributions are parameterized and incorporated into the pressure sensor model to establish the EMI environment. Based on this coupled model, the changes in the graphene membrane parameters under electromagnetic interference are analyzed. Finally, these parameter variations are introduced into the micro-pressure sensor model to evaluate the impact of EMI on the sensor’s output signal.

**Figure 2:**
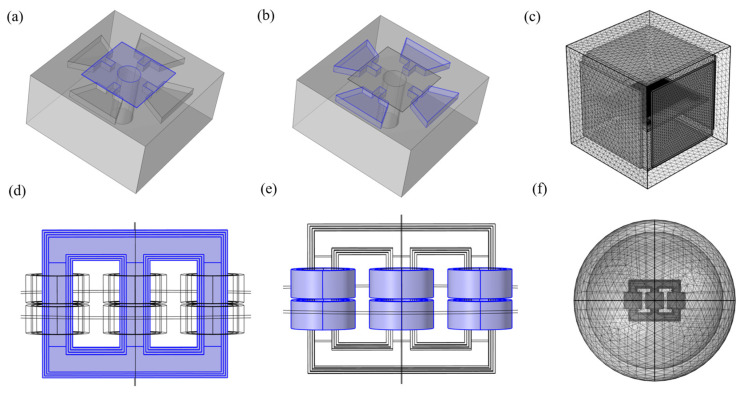
Modeling of nanoelectromechanical pressure sensor and three-phase transformers. (**a**) Model of the Sensor’s Sensitive Membrane: Graphene; (**b**) Four metal electrodes of the sensor; (**c**) Simulation modeling of sensors operating in an EMI environment; (**d**) The iron core of three-phase transformer; (**e**) Primary coil and secondary collar of three-phase transformer. (**f**) Transformer Simulation Spatial Model.

Accurate modeling of the sources of electromagnetic interference (EMI) is essential when studying its effects on motor drive systems. This paper presents a methodology for modeling and simulating electromagnetic interference (EMI) sources in a system based on a three-phase transformer. Figure 2 illustrates the modeling process: Figure 2a shows a schematic of the graphene structure; Figure 2b depicts the modeling of the four electrodes of the sensor; Figure 2c presents the sensor placed within the electromagnetic environment; Figure 2d–f show the modeling of the S7-315/10 three-phase transformer (Chuanglian Huitong Electrical Equipment Co., Ltd., Beijing, China) using the electromagnetic module of COMSOL Multiphysics 6.1. The core of a three-phase transformer consists of thin laminated sheets of silicon steel and serves as the magnetic circuit, concentrating and guiding the magnetic flux while minimizing energy losses, as shown in Figure 2d,e. The transformer used in this study adopts a core-type structure, in which the windings are arranged around central magnetic core. This design provides a closed magnetic path for the flux generated by the windings, improving magnetic efficiency and minimizing leakage. The windings are made of insulated copper conductors and are divided into primary and secondary coils to achieve voltage transformation through electromagnetic induction. In practical transformers, additional components such as insulating materials, transformer oil, cooling systems, and tap changers are used for insulation, cooling, and voltage regulation. However, the primary objective of this study is to analyze the spatial distribution of electric and magnetic fields in the working region of a three-phase transformer rather than thermal behavior or insulation performance. Therefore, components such as insulating structures, transformer oil, and cooling systems are not included in the simulation model. From an electromagnetic perspective, the field distribution in the surrounding space is mainly governed by the current in the windings and the magnetic permeability of the core, whereas auxiliary materials such as transformer oil and insulation typically exhibit relative permeability close to unity and limited contrast in dielectric properties. As a result, their influence on the overall electromagnetic field distribution is comparatively weak. Similar simplifications have been widely adopted in transformer electromagnetic field simulations to reduce computational complexity while maintaining sufficient accuracy. Therefore, in this study, only the core, primary windings, and secondary windings of the three-phase transformer are modeled and simulated. The modeling process of the corresponding regions is shown in Figure 2d–f. The input voltage of the simulation model is set to 0.4 kV, and the electric and magnetic field distributions at different spatial locations around the transformer can be obtained through simulation. This simplified modeling approach represents a reasonable trade-off between computational efficiency and simulation accuracy.

## 3. Results and Discussion

### 3.1. Nanoelectromechanical Pressure Sensor Stimulation Result

**Figure 3:**
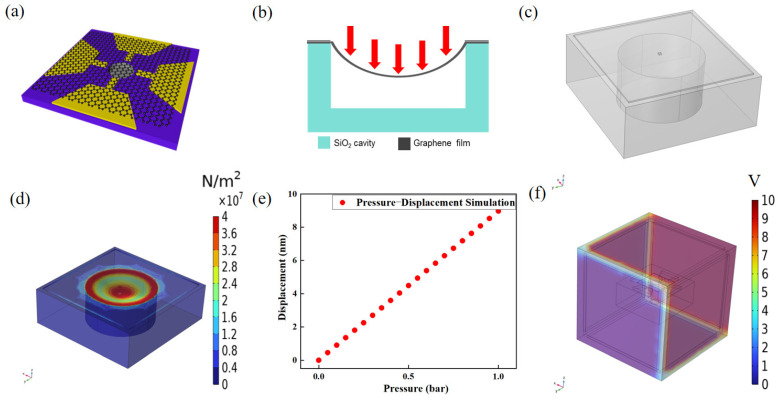
The working principle of the nanoelectromechanical pressure sensor based on suspended graphene. (**a**) Structural diagram of the pressure sensor; (**b**) Working principle of the pressure sensor; (**c**) Simplified Model of pressure sensor in COMSOL Software Simulation; (**d**) Simulation Results of Graphene Film Deformation for pressure Sensors; (**e**) Primary coil and secondary collar of three-phase transformer; (**f**) Simulation Results of Performance Changes in pressure sensors Under Electromagnetic Interference (EMI) Environment.

Figure 3a shows the structure diagram of the nano-electromechanical pressure sensor. In our simulation, graphene membranes are suspended over cavities etched into a SiO_2_ film on a silicon substrate. The graphene is directly contacted with metal electrode, and the devices are wire-bonded into a chip package. If a pressure difference exists between the inside and outside of the cavity, the graphene membrane sealing the cavity is deflected and strained. This deformation results in a change in the device’s resistivity due to the piezoresistive effect in the graphene. To estimate the piezoresistive gauge factor of the graphene transducer in our pressure sensor, the change in resistance of the cavity region must be determined. The simulation parameters presented here utilize the pertinent data studied by Shou-En Zhu et al. [7]. A finite element analysis of the deflection was conducted using COMSOL Multiphysics 6.1. The resistance of graphene at different pressures was simulated. In the simulation, the resistance change in the graphene film was determined based on the displacement of the graphene film combined with the piezoresistive effect, as described later in Equation (1). The displacement of the graphene film after compression can be determined by simulation analysis. The graphene exhibits strong adhesion to SiO_2_ substrates [32] and is nearly impermeable to gases [33]. As a result, the cavity in the simulated structure remains permanently sealed [34].

In this study, the cavity pressure is assumed to be constant and equal to atmospheric pressure, and gas dynamic effects are neglected. This assumption is justified by the small deformation regime of the suspended graphene membrane. The maximum central displacement obtained from simulation is on the nanometer scale, which is several orders of magnitude smaller than the cavity dimensions. As a result, the corresponding volume change in the sealed cavity is negligible, and the internal pressure variation can be reasonably ignored. In addition, graphene membranes are known to be nearly impermeable to gases, ensuring that the cavity remains effectively sealed. Under such conditions, gas compression effects introduce only minor perturbations compared to the dominant electromechanical response of graphene. Therefore, this simplification is considered reasonable for analyzing the pressure-induced deformation and EMI-induced electrical response of the graphene sensor, while significantly reducing model complexity. The nanoelectromechanical pressure sensor function described here primarily relies on a suspended graphene and cavity structure. The graphene is electrically connected to the metal electrodes. The sensor’s substrate is Silicon-on-Insulator (SOI) wafer. The sensor’s simulation model has been simplified to the structure shown in Figure 3b. When a pressure difference exists between the inside and outside of the cavity, the graphene membrane sealing the cavity is deflected and strained. This deformation results in a change in the device’s resistivity due to the piezoresistive effect in graphene. In the simulation, a gradient pressure was applied to the upper surface of the graphene film, and the operating curve of the graphene pressure sensor was obtained, as shown in Figure 3e. The simulation results illustrate the output currents of the graphene pressure sensor under different pressure states at a fixed bias voltage. In subsequent EMI environments, the sensor’s output current is perturbed. The source of this perturbation will be analyzed in the EMI simulation section.

The calculation of the pressure difference serves as a foundation for simulations in COMSOL Multiphysics, providing essential data support and initial values for resistance variation calculations. It is assumed that the gases are under standard pressure and pressure conditions. The pressure range applied on the pressure sensor is 0–1 bar. Additionally, the risk of membrane rupture or graphene delamination from the SiO_2_ surface at the membrane edge, which has been reported to occur at pressures in the MPa, can be reasonably disregarded in this study [35]. The strain of the suspended graphene film under compression can be simulated using commercial COMSOL software, as illustrated in Figure 2c and Figure 3b. The simulations utilize the solid mechanics module of COMSOL software version 6.1. The relationship between the displacement of the suspended graphene center in the simulation model and the applied pressure is expressed in Equation (1) [36].

(1) $$\Delta p=\frac{128Et{d\delta }^{3}}{3(1-v)a^{4}}+\frac{16\delta_{0}t\delta^{3}}{a^{2}}$$

The Δp is the pressure difference in the graphene film suspended on the cavity, E is the Young’s modulus, v is the Poisson’s ratio, t is the film thickness, a is the square cavity width, d is the maximum Center Deflection of the Diaphragm and $\sigma_{0}$ is the initial film stress, δ is the maximum vertical displacement of the center point. The simulation results are presented in Figure 3d,e. The observed variation in resistance across pressure sensors (Figure 3e) primarily arises from the piezoresistive effect inherent to graphene. The piezoresistive effect of graphene is a fundamental principle underpinning the operation of this pressure sensor. Therefore, the theoretical calculations related to the piezoresistive effect of suspended graphene in this sensor are conducted. The piezoresistive effect of graphene can be expressed by an equation, as detailed later in Equation (2) [37]. Combined with the simulation results in COMSOL, the formula is simplified by extracting the simulation parameters and then calculated in MATLAB 2024b.

(2) $$R=\rho \frac{L}{W_{t}}=\frac{1}{2qN_{e}\mu_{e}}\cdot \frac{\left(1+\varepsilon_{xx}\right)}{\left(1+\varepsilon_{yy}\right)}\cdot \frac{L}{W_{t}}$$

The ρ is the resistivity of the membrane region, $N_{e}$ is the electron density, and $\mu_{e}$ is the electron mobility. $\varepsilon_{xx\;}\;$and$\;\varepsilon_{yy}$ are strain components in the x and y directions of the membrane region. Furthermore, *q* is the electronic charge, *L* and $W_{t}$ represent the effective length and width of the graphene film, which vary in the directions parallel and perpendicular to the applied stress, respectively. This equation shows that the resistance of the graphene film depends not only on its intrinsic material properties ($\rho ,N_{e},\mu_{e}$) but also on its geometrical changes induced by strain ($\varepsilon_{xx},\varepsilon_{yy},L,W_{t}$).

The simulation results are presented in Figure 3d,e. It can be observed that the displacement of the suspended graphene center exhibits excellent linearity with applied stress, with a maximum central displacement of 9 nm. The maximum displacement observed in this simulation is well below the upper limit of graphene’s elastic recovery stretchability [38]. The simulation result in Figure 3f confirms the voltage distribution under EMI, and a more comprehensive discussion of the graphene film’s mechanical durability can be found in Section 3.3.

### 3.2. EMI Environment Stimulation Result

Graphene thin-film resistors inherently generate noisy signals due to conduction coupling and radiation coupling when the nanoelectromechanical pressure sensor is in a complex EMI environment. Here, conduction coupling refers to noise transmitted through physical electrical connections or wires, while radiation coupling refers to noise transmitted through electromagnetic waves directly interacting with the chip, which generally has a greater impact. Since conduction coupling can be effectively reduced by external circuits, this work focuses on exploring the impact of radiation coupling on the graphene sensing mechanism. After completing the simulation of the sensor’s working principle, it is necessary to set up the electromagnetic interference environment. The first step in modeling the nanoelectromechanical pressure sensor EMI environment is to determine the direction and magnitude of the interference source.

**Figure 4:**
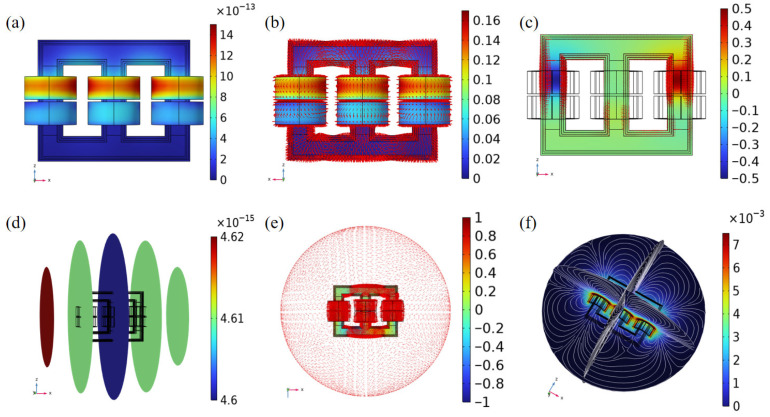
Simulation Results of Electromagnetic interference source of a Three-Phase Transformer Based on COMSOL. (**a**) Electric Field Distribution (C/m^2^); (**b**) Electric Field and Power Density Distribution (V/m); (**c**) Three-phase transformer current distribution (A/m^2^); (**d**) Electric field distribution on a cross-sectional plane (V/m); (**e**) Three-phase transformer magnetic field distribution effect (A/m); (**f**) Transformer space magnetic flux density (T).

According to the simulation results of the three-phase transformer in Figure 4, combined with the relevant parameters in the electrical safety manual, we set the electromagnetic interference environment of the sensor at a distance of 1 m from the three-phase transformer. Based on the electric field distribution shown in Figure 4a, the electric field intensity at a distance of 1 m from the three-phase transformer is measured as 0.325 kV/m. According to the simulation model of the pressure sensor sensitivity principle, it is evident that the interference from the electromagnetic environment on the operational state of the graphene-sensitive membrane primarily occurs by altering the carrier transmission efficiency. Analyzing the magnitude, variation trends, and interplay of electromagnetic parameters—including electric field intensity, energy density, current density, magnetic field intensity, and magnetic flux—during three-phase transformer operation provides critical theoretical insights for designing controlled electromagnetic interference (EMI) environments.

The EMI environment is modeled in COMSOL Multiphysics 6.1. and the variation in graphene’s electrical properties under this environment is simulated and analyzed, as shown in Figure 4. Due to the MEMS sensor’s significantly smaller scale on the micrometer level compared to the three-phase transformer, which operates on the meter level, it can be modeled as a point source in the electromagnetic interference (EMI) analysis, with the three-phase transformer acting as the primary EMI source. The simulated electromagnetic field distribution of the three-phase transformer is shown in Figure 4. Figure 4a illustrates the electric displacement field distribution under the three-phase transformer. Analysis of the simulated power-density (Figure 4b) and current-density (Figure 4c) distributions reveals that the secondary coil exhibits the highest concentration of electrical energy during operation. The electric displacement field distribution in Figure 4d enables determination of the magnitude and direction of the electric field at a distance of 1 m from the three-phase transformer, providing a theoretical foundation for defining key parameters of the spatially varying electric field. Figure 4e,f depict the magnetic field and magnetic flux distributions under the three-phase transformer’s operating conditions. A comparative analysis with the electric displacement field data in Figure 4d reveals that the magnetic field intensity is significantly weaker than the electric field intensity. Consequently, for modeling the sensor’s electromagnetic interference (EMI) environment, this study focuses exclusively on the three-phase transformer’s electric field effects, neglecting the magnetic component due to its negligible magnitude.

The COMSOL three-phase transformer simulation leverages the Magnetostatics interface within the AC/DC Module to model steady-state magnetic fields, neglecting displacement currents (∂D/∂t ≈ 0) for low-frequency operating conditions. The pressure sensor simulation utilizes the Solid Mechanics Module to analyze structural deformation and stress. Due to the absence of a Multiphysics coupling mechanism between the AC/DC Module and Solid Mechanics Module in the COMSOL, these simulations cannot be integrated into a unified model without introducing explicit cross-domain interactions. Therefore, eight spatial electrodes can be used to simulate the electric field interference signal generated by the three-phase transformer. By combining the Lorentz force theory with the stimulation result in Figure 4, it can be observed that the magnetic field has a negligible effect on the carriers compared to the electric field. The Lorentz force equation describes the force experienced by a charged particle moving through both electric and magnetic fields, and is given by:

(3) $$\vec{F}=q(\vec{E}+\vec{v}\times \vec{B})$$

where F is magnitude of the Lorentz force. E is the electric field. v is magnitude of the velocity of the particle. B is magnitude of the magnetic field. Simulating the electromagnetic interference (EMI) environment generated by the three-phase transformer, a spatial electric field is applied in the sensor’s simulation module. Three pairs of electrode plates were set in pressure sensor simulation model to construct parallel electric field simulated by three-phase transformer. Three pairs of electrode plates are positioned along the X, Y, and Z axes of the coordinate system, with the pressure sensor’s central space as the origin, as shown in Figure 2c. Based on the vector operation principles, varying the potentials of the electrodes along different coordinate axes allows the creation of a spatial electric field with arbitrary direction and magnitude. This approach enables the emulation of the electromagnetic interference (EMI) environment experienced by the graphene sensor, corresponding to the external fields generated by the three-phase transformer. In this way, the transformer simulation results are translated into a controllable laboratory-scale electrode setup. Figure 3f illustrates the resulting simulated EMI environment created by the spatial electrode configuration, providing a bridge between the transformer field simulations and the sensor response analysis.

### 3.3. The Baseline Drift of Pressure Sensor in EMI Environments

The sensitivity of graphene-based pressure sensors is governed by the mobility and density of carriers. In electromagnetic interference (EMI)-prone environments, these carrier properties are perturbed, resulting in unstable sensor signal outputs. Reductions in carrier mobility result in a significant increase in electrical resistance. This resistance increase persists in strained graphene due to electrostatic equilibrium between interfacial charge dipoles—specifically, those formed at the graphene-top surface and graphene-substrate interfaces. Crucially, since these sensors operate by converting mechanical pressure into electrical signals through modulated carrier transport rates, EMI disrupts the force-to-signal transduction mechanism. Consequently, EMI compromises the accuracy and reliability of graphene-based pressure sensors in real-world electromagnetic environments. Two key indicators reflecting such accuracy and reliability degradation are Signal-to-Noise Ratio (SNR) and baseline drift: SNR refers to the ratio of the effective power (or amplitude) of the useful signal output by the sensor to the power (or amplitude) of the background noise, usually expressed in decibels (dB) as

(4) $$SNR(dB)=10\log_{10}(\frac{P_{signal}}{P_{noise}})$$

where $P_{signal}$ is the effective power of the useful signal and $P_{noise}$ is the effective power of background noise; baseline drift refers to the slow deviation of the sensor’s steady-state output signal from its initial reference baseline, quantified by %FS as

(5) $$BaselineDrift(\&FS)=\frac{\left|V_{drift}-V_{base}\right|}{V_{FS}}\times 100\%$$

where $V_{drift}$ is the output voltage under interference, $V_{base}$ is the initial baseline voltage, and $V_{FS}$ is the full-scale output voltage.

**Figure 5:**
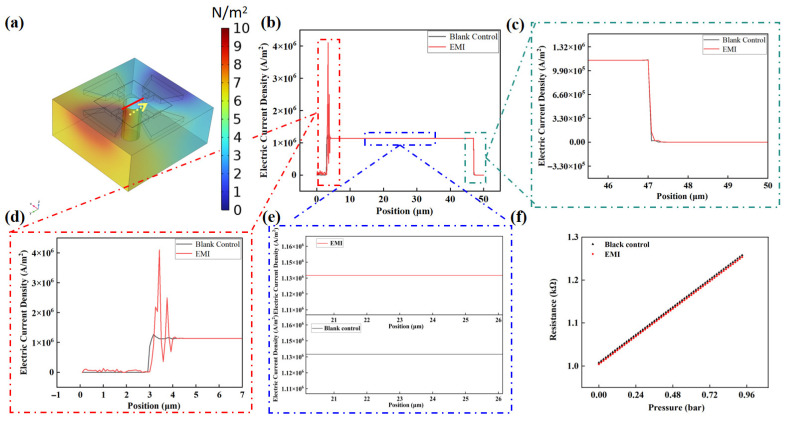
Simulation results of the signal variation in the sensor’s sensitive film under EMI conditions. (**a**) Schematic diagram of sensor sensitive film data acquisition area; (**b**) Simulation results of current density distribution of sensor sensitive film under electromagnetic interference; (**c**–**e**) A partial enlarged view of (**b**); (**f**) Error analysis of output signal of pressure sensor in electromagnetic interference environment.

Figure 5 presents the simulation results of the suspended graphene-based pressure sensor under electromagnetic interference (EMI). Figure 5a illustrates the electric field distribution across the sensor surface during EMI exposure. The red short line region in Figure 5a corresponds to the graphene film’s current density area, as analyzed in subsequent Figure 5b. To quantitatively evaluate the local electrical response, the electric current density was sampled along the characteristic path indicated by the yellow curve in Figure 5a. Furthermore, the ‘Blank Control’ represents the intrinsic electrical state of the device in a zero-interference environment, serving as a baseline for the EMI-affected results. Figure 5b–d provide magnified views of the graphene current density curves under varying conditions. Figure 5c focuses on the region near the sensor’s negative electrode, where the current density curve exhibits minimal fluctuation (≤2% deviation from baseline) before and after EMI exposure. In contrast, Figure 5d highlights the graphene current density near the positive electrode, where EMI induces significant fluctuations. Here, the current density near critical graphene regions shows peak fluctuations of up to 350%, with the magnitude increasing toward the electrode edge. The 5% fluctuation arises primarily from EMI-induced currents and carrier density modulation, influenced by graphene’s high conductivity and electron mobility. Detailed characterization of EMI parameters (frequency, intensity) and graphene film properties would further elucidate the dominant mechanisms. Figure 5e demonstrates that graphene regions far from both electrodes remain largely unaffected by EMI, with current density fluctuations remaining negligible. The sensitivity of graphene-based pressure sensors is governed by the mobility and density of graphene carriers. In environments with electromagnetic interference (EMI), these carrier properties are perturbed, resulting in unstable sensor signal outputs. The carrier mobility of graphene is influenced by substrate interactions [39], contact resistance [40], and quantum effects [41], and the carrier transport behavior of graphene is a result of the combined effects of these factors. This paper primarily investigates the impact of the electric field on the carrier mobility of graphene, with the assumption that graphene is considered in an ideal state. Here, we mainly study the effect of the external electric field, specifically the electromagnetic interference environment, on the carrier mobility of graphene. The possible reason is that EMI enhances impurity scattering in the graphene, which is directly in contact with the metal electrode in SiO_2_ [42]. Consequently, the mobility of graphene on SiO_2_ is limited by impurity scattering. As a result, EMI introduces noise into the graphene film’s resistance measurements, leading to distortion. Moreover, impurity scattering in the suspended graphene structure is significantly reduced, resulting in lower susceptibility to EMI in the suspended region. Since graphene pressure sensors operate by translating pressure-induced mechanical strain into electrical signals via modulated carrier transport rates, EMI disrupts this critical force-to-electrical signal conversion process. Consequently, EMI compromises the accuracy and reliability of graphene-based pressure sensors in electromagnetically noisy environments. The simulation results reveal pronounced fluctuations in current density at the graphene-metal electrode interface following electromagnetic interference (EMI) exposure. Notably, suspended graphene demonstrates markedly superior EMI resilience compared to its substrate-supported counterpart. Following the analytical framework established in Equations (6) and (7), the electromagnetic interference (EMI)-induced fluctuations in the graphene film’s current density are systematically mapped to variations in the output resistance of the pressure sensor in Figure 5f. Here, J represents the current density in the graphene film.

(6) $$\mu =\frac{J}{nqE}$$

(7) $$R=\rho \frac{L}{W_{t}}=\frac{E}{2J}\cdot \frac{\left(1+\varepsilon_{xx}\right)}{\left(1+\varepsilon_{yy}\right)}\cdot \frac{L}{W_{t}}$$

While the resistance response of graphene is theoretically governed by its intrinsic relationship (Equation (2)), directly quantifying EMI-induced deviations in the pressure-resistance curve of the graphene sensor using this formula is impractical. The phenomenon arises because of EMI electron mobility and carrier density—critical variables in Equation (2)—cannot be reliably extracted from first-principles simulations. To address this, we derive a modified framework (Equation (7)) by integrating Equation (6) with the fundamental piezoresistive relationships of graphene, enabling indirect analysis of EMI’s impact on the sensor’s electromechanical behavior. Leveraging the theoretical framework of Equation (2), the post-EMI output characteristics of the pressure sensor were computationally modeled using MATLAB software, with the results quantitatively illustrated in Figure 5d. The fig juxtaposes the sensor’s electromechanical response under EMI (red curve) against its baseline performance in an interference-free environment (black curve). The simulations reveal that a baseline drift (>5% FS) was produced in graphene current density under EMI, which elevates signal-to-noise ratio (SNR) degradation exceeding 15 dB, highlighting the interplay between electromagnetic perturbations and the sensor’s functional stability.

## 4. Conclusions

This study presents a multiphysics simulation framework to investigate the impact of electromagnetic interference (EMI) on graphene-based piezoresistive pressure sensors, using a three-phase transformer as a representative EMI source. The simulation integrates electromagnetic field distribution with the electromechanical response of a suspended graphene membrane, enabling a systematic analysis of EMI-induced perturbations in sensor output. The results demonstrate that electromagnetic interference can significantly affect the electrical response of graphene-based sensors by modulating carrier transport properties. Under EMI conditions, the graphene current density exhibits noticeable spatial non-uniformity, particularly near the graphene–metal electrode interfaces. This leads to observable signal instability, including baseline drift on the order of ~5% full scale (FS) and signal-to-noise ratio (SNR) degradation to values over ~15 dB under typical simulation conditions. These effects are attributed to enhanced carrier scattering, parasitic coupling, and local electric-field concentration. Furthermore, the comparison between substrate-supported and suspended graphene configurations reveals that suspended graphene structures exhibit improved resistance to electromagnetic interference. This enhancement is mainly attributed to reduced substrate-induced scattering and weakened parasitic coupling, resulting in more stable carrier transport under external electric-field perturbations. The results indicate that structural design plays a critical role in improving the electromagnetic compatibility (EMC) of graphene-based sensors. Although this study is based on numerical simulations, the observed trends are consistent with previously reported behaviors of graphene under external electric-field perturbations, including mobility modulation and noise enhancement. In addition, sensitivity analysis of key material parameters, such as carrier mobility and initial conductivity, indicates that while absolute values of signal variation may change, the overall trends of EMI-induced degradation remain consistent. This suggests that the conclusions are robust with respect to reasonable parameter variations. It should be noted that the present model is intended to capture the relative variation trends and underlying physical mechanisms rather than provide quantitatively exact predictions. Future work will focus on incorporating more detailed transport models and experimental validation to further improve the accuracy and applicability of the proposed framework. Overall, this work provides a theoretical basis for understanding EMI effects in graphene-based pressure sensors and offers design insights for enhancing their performance in complex electromagnetic environments, such as power systems and industrial IoT applications.

## Data Availability

The data that support the findings of this study are available from the corresponding author upon reasonable request.

## Abbreviations

The following abbreviations are used in this manuscript:

EMI	Eletromagnetic Interference
SNR	Signal-to-Noise Ratio
EMC	Eletromagnetic Compatibility
LoT	Internet of Things
FS	Full Scale
SOI	Sillicon-on-Insulator
CETC	China Electronics Technology Group Corporation
